# Bis(μ-2,2′-{[4-(carb­oxy­meth­oxy)phen­yl]aza­nedi­yl}diacetato)­bis­[(1,10-phenanthroline)copper(II)]

**DOI:** 10.1107/S1600536810048488

**Published:** 2010-11-27

**Authors:** Yan Zhao, Tonghen Pan, Zhitao Chen

**Affiliations:** a College of Life Science, Fujian Agriculture and Forestry University, Fuzhou, Fujian 350002, People’s Republic of China; b Department of Chemistry, Fuzhou University, Fuzhou, Fujian 350108, People’s Republic of China; c Fuqing Entry–exit Inspection and Quarantine Bureau, Fuqing, Fujian 350300, People’s Republic of China

## Abstract

The crystal structure of the binuclear title compound, [Cu_2_(C_12_H_11_NO_7_)_2_(C_12_H_8_N_2_)_2_], consists of a complex mol­ecule, which lies about a crystallographic inversion centre with one half-mol­ecule in the asymmetric unit. The Cu^II^ cation is bonded to three N atoms and three O atoms, in a Jahn–Teller-distorted octa­hedral geometry. The basal plane is defined by the two N atoms from the 1,10-phenathroline and two deprotonated O atoms of the polycarboxyl­ate ligand. The axial positions are occupied by the azane N atom and a bridging carboxyl­ate O atom from the second polycarboxyl­ate ligand. The complex mol­ecules are linked through O—H⋯O hydrogen bonds into extended chains running parallel to [010].

## Related literature

For general background to the applications of polycarboxyl­ate ligands, see: Ghermani *et al.* (1994 ▶); Ruiz-Perez *et al.* (2000 ▶); Ye *et al.* (2005 ▶); Kido *et al.* (2003 ▶). For the features of flexible multidentate aromatic polycarboxyl­ate ligands, see: Wang *et al.* (2009 ▶); Pan *et al.* (2008 ▶); Dong *et al.* (2006 ▶).
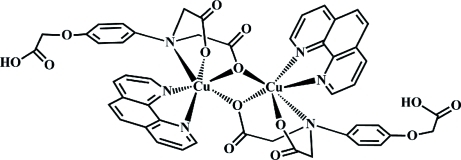


## Experimental

### 

#### Crystal data

[Cu_2_(C_12_H_11_NO_7_)_2_(C_12_H_8_N_2_)_2_]
*M* *_r_* = 1049.92
Monoclinic, 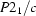

*a* = 8.7410 (17) Å
*b* = 10.886 (2) Å
*c* = 22.239 (4) Å
β = 90.85 (3)°
*V* = 2115.8 (7) Å^3^
*Z* = 2
Mo *K*α radiation
μ = 1.09 mm^−1^
*T* = 293 K
0.26 × 0.18 × 0.12 mm

#### Data collection

Rigaku Mercury CCD area-detector diffractometer
Absorption correction: multi-scan (*RAPID-AUTO*; Rigaku, 1998 ▶) *T*_min_ = 0.85, *T*_max_ = 1.00
14139 measured reflections
3604 independent reflections
3408 reflections with *I* > 2σ(*I*)
*R*_int_ = 0.052

#### Refinement

*R*[*F* ^2^ > 2σ(*F* ^2^)] = 0.059
*wR*(*F* ^2^) = 0.103
*S* = 1.25
3604 reflections
317 parameters
H-atom parameters constrained
Δρ_max_ = 0.27 e Å^−3^
Δρ_min_ = −0.35 e Å^−3^

### 

Data collection: *CrystalClear* (Rigaku, 2002 ▶); cell refinement: *CrystalClear*; data reduction: *CrystalClear*; program(s) used to solve structure: *SHELXS97* (Sheldrick, 2008 ▶); program(s) used to refine structure: *SHELXL97* (Sheldrick, 2008 ▶); molecular graphics: *SHELXTL* (Sheldrick, 2008 ▶); software used to prepare material for publication: *SHELXL97*.

## Supplementary Material

Crystal structure: contains datablocks I, global. DOI: 10.1107/S1600536810048488/sj5063sup1.cif

Structure factors: contains datablocks I. DOI: 10.1107/S1600536810048488/sj5063Isup2.hkl

Additional supplementary materials:  crystallographic information; 3D view; checkCIF report

## Figures and Tables

**Table:** 

Cu1—O7	1.987 (3)
Cu1—O5	1.997 (3)
Cu1—N2	2.003 (3)
Cu1—N3	2.049 (3)
Cu1—O7^i^	2.293 (3)
Cu1—N1	2.460 (3)

**Table:** 

O7—Cu1—O5	92.08 (11)
O7—Cu1—N2	171.29 (12)
O5—Cu1—N2	93.61 (12)
O7—Cu1—N3	91.96 (12)
O5—Cu1—N3	170.40 (12)
N2—Cu1—N3	81.44 (13)
O7—Cu1—O7^i^	77.11 (11)
O5—Cu1—O7^i^	93.01 (10)
N2—Cu1—O7^i^	109.13 (11)
N3—Cu1—O7^i^	96.38 (11)
O7—Cu1—N1	74.69 (11)
O5—Cu1—N1	76.84 (11)
N2—Cu1—N1	100.21 (12)
N3—Cu1—N1	95.87 (12)
O7^i^—Cu1—N1	149.54 (10)

Symmetry code: (i) 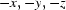.

**Table 2:** Hydrogen-bond geometry (Å, °)

*D*—H⋯*A*	*D*—H	H⋯*A*	*D*⋯*A*	*D*—H⋯*A*
O2—H2⋯O4^ii^	0.82	1.82	2.622 (4)	164

Symmetry code: (ii) 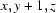.

**Fig. 1.**
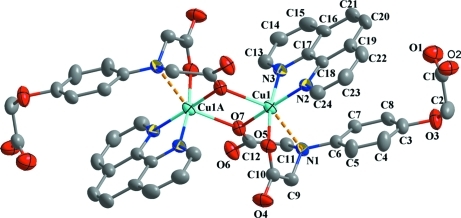
The structure of the title compound with 30% probability displacement ellipsoids. The weak axial Cu—N bonds are shown as dashed lines. H atoms have been omitted for clarity. [Atoms labelled with the suffix A are related to other atoms by the symmetry code: [-x,-y,-z]

**Fig. 2.**
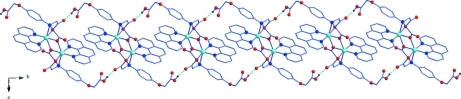
A view of the hydrogen-bonded 1-dimensional chains running parallel to [010]. The hydrogen bonds are shown as dashed lines.

## supplementary crystallographic information

### Comment

Carboxylate-metal coordination compounds have received considerable attention due to their potential applications in catalysis and pharmaceutical chemistry (Ghermani *et al.*, 1994; Ruiz-Perez *et al.*, 2000), molecular recognition and magnetic materials (Ye *et al.*, 2005); Kido, *et al.*, 2003). In recent years, several studies have focused on flexible multidentate aromatic polycarboxylate ligands, because of their remarkable features. These ligands contain carboxylate groups, which can provide a variety of coordination modes (Wang *et al.*, 2009). They also offer the opportunity to form hydrogen bonds leading to supramolecular structures (Pan *et al.*, 2008). Furthermore, such ligands can be used to construct unprecedented topological frameworks (Dong *et al.*, 2006). Here, we present the structure of the title compound (I), a copper complex with 2,2'-(4-(carboxymethoxy)phenylazanediyl)diacetate, a flexible multidentate aromatic polycarboxylate ligand.

As shown in Fig. 1, the binuclear complex contains two Cu^II^ cations with very distorted octahedral geometries. The basal plane of each coordination site is defined by the N2 and N3 atoms from the 1,10-phenathroline ligand and the deprotanated O5 and O7 atoms from a polycarboxylate ligand. The axial positions are occupied by the azane N1 atom and a bridging O7A atom from the second polycarboxylate ligand. The angle O7A—Cu1—N1 and the axial bond lengths are respectively 149.54 (10)°; Cu1—O7A, 2.293 (3)Å; Cu1—N1, 2.460 (3)Å which demonstrate a very distorted octahedral coordination geometry due to the Jahn-Teller effect. The packing is stabilized through intermolecular hydrogen-bonding between the uncoordinated carboxyl O—H group and a neighboring carbonyl oxygen atom. This results in a 1-dimensional hydrogen-bonded chain parallel to the [010] direction (Fig. 2 and Table 1).

### Experimental

The polycarboxylate ligand (0.082 g, 0.3 mmol), Cu(CH_3_COO)_2_.2H_2_O (0.044 g, 0.2 mmol) and 1,10-phenathroline (0.055 g, 0.3 mmol) were dissolved in a mixed solvent of ethanol and water (8 ml, 5:3 *v*/*v*) and stirred for 4 h at room temperature. The mixture was filtered and allowed to evaporate in air at room temperature. Block-like blue crystals separated from the filtrate after 8 days.

### Refinement

The H2 atom bound to O2 was placed in an idealized position in the riding-model approximation with O—H = 0.82 Å, All other H atoms were placed in calculated positions with a C—H bond distance of 0.93 Å and *U*_iso_(H) = 1.2Ueq of the parent atoms.

### Crystal data

**Table:**

[Cu_2_(C_12_H_11_NO_7_)_2_(C_12_H_8_N_2_)_2_]	*F*(000) = 1076
*M**_r_* = 1049.92	*D*_x_ = 1.648 Mg m^−^^3^
Monoclinic, *P*2_1_/*c*	Mo *K*α radiation, λ = 0.71073 Å
Hall symbol: -P 2ybc	Cell parameters from 25 reflections
*a* = 8.7410 (17) Å	θ = 12–18°
*b* = 10.886 (2) Å	µ = 1.09 mm^−^^1^
*c* = 22.239 (4) Å	*T* = 293 K
β = 90.85 (3)°	Block, blue
*V* = 2115.8 (7) Å^3^	0.26 × 0.18 × 0.12 mm
*Z* = 2	

### Data collection

**Table:**

Rigaku Mercury CCD area-detector diffractometer	3604 independent reflections
Radiation source: fine-focus sealed tube	3408 reflections with *I* > 2σ(*I*)
graphite	*R*_int_ = 0.052
ω scans	θ_max_ = 24.7°, θ_min_ = 3.1°
Absorption correction: multi-scan (*RAPID-AUTO*; Rigaku, 1998)	*h* = −10→10
*T*_min_ = 0.85, *T*_max_ = 1.00	*k* = −12→12
14139 measured reflections	*l* = −26→26

### Refinement

**Table:**

Refinement on *F*^2^	Primary atom site location: structure-invariant direct methods
Least-squares matrix: full	Secondary atom site location: difference Fourier map
*R*[*F*^2^ > 2σ(*F*^2^)] = 0.059	Hydrogen site location: inferred from neighbouring sites
*wR*(*F*^2^) = 0.103	H-atom parameters constrained
*S* = 1.25	*w* = 1/[σ^2^(*F*_o_^2^) + (0.0128*P*)^2^ + 3.5126*P*] where *P* = (*F*_o_^2^ + 2*F*_c_^2^)/3
3604 reflections	(Δ/σ)_max_ = 0.001
317 parameters	Δρ_max_ = 0.27 e Å^−^^3^
0 restraints	Δρ_min_ = −0.35 e Å^−^^3^

### Special details

**Table:**

Geometry. All e.s.d.'s (except the e.s.d. in the dihedral angle between two l.s. planes) are estimated using the full covariance matrix. The cell e.s.d.'s are taken into account individually in the estimation of e.s.d.'s in distances, angles and torsion angles; correlations between e.s.d.'s in cell parameters are only used when they are defined by crystal symmetry. An approximate (isotropic) treatment of cell e.s.d.'s is used for estimating e.s.d.'s involving l.s. planes.
Refinement. Refinement of *F*^2^ against ALL reflections. The weighted *R*-factor *wR* and goodness of fit *S* are based on *F*^2^, conventional *R*-factors *R* are based on *F*, with *F* set to zero for negative *F*^2^. The threshold expression of *F*^2^ > σ(*F*^2^) is used only for calculating *R*-factors(gt) *etc*. and is not relevant to the choice of reflections for refinement. *R*-factors based on *F*^2^ are statistically about twice as large as those based on *F*, and *R*- factors based on ALL data will be even larger.

### Fractional atomic coordinates and isotropic or equivalent isotropic displacement parameters (Å^2^)

**Table:**

	*x*	*y*	*z*	*U*_iso_*/*U*_eq_	
Cu1	0.05701 (5)	0.10839 (4)	0.04897 (2)	0.03031 (15)	
O5	−0.0341 (3)	0.0219 (2)	0.11910 (12)	0.0356 (7)	
O7	0.1363 (3)	−0.0478 (2)	0.01526 (12)	0.0326 (6)	
N1	0.2819 (4)	0.0561 (3)	0.11173 (14)	0.0304 (7)	
N2	0.0092 (4)	0.2758 (3)	0.08106 (15)	0.0319 (8)	
N3	0.1739 (4)	0.2109 (3)	−0.01248 (15)	0.0330 (8)	
O6	0.3412 (3)	−0.1600 (3)	−0.00516 (14)	0.0459 (8)	
O2	0.1572 (4)	0.7030 (3)	0.24279 (14)	0.0528 (9)	
H2	0.1176	0.7503	0.2184	0.063*	
O3	0.4623 (4)	0.4841 (3)	0.23479 (15)	0.0550 (9)	
O1	0.3190 (4)	0.6653 (3)	0.16736 (15)	0.0605 (9)	
O4	−0.0057 (3)	−0.1359 (3)	0.18195 (14)	0.0500 (8)	
C1	0.2762 (5)	0.6474 (4)	0.2177 (2)	0.0434 (11)	
C2	0.3508 (6)	0.5602 (4)	0.2620 (2)	0.0546 (13)	
H2A	0.3991	0.6073	0.2941	0.066*	
H2B	0.2728	0.5089	0.2798	0.066*	
C3	0.4095 (5)	0.3800 (4)	0.2047 (2)	0.0401 (10)	
C4	0.2869 (5)	0.3104 (4)	0.2226 (2)	0.0482 (12)	
H4	0.2304	0.3347	0.2557	0.058*	
C5	0.2476 (5)	0.2047 (4)	0.19176 (19)	0.0432 (11)	
H5	0.1638	0.1593	0.2043	0.052*	
C6	0.3299 (4)	0.1642 (4)	0.14239 (17)	0.0291 (9)	
C7	0.4531 (4)	0.2351 (4)	0.12463 (18)	0.0335 (9)	
H7	0.5100	0.2110	0.0917	0.040*	
C8	0.4928 (5)	0.3423 (4)	0.15567 (19)	0.0377 (10)	
H8	0.5759	0.3888	0.1432	0.045*	
C9	0.2205 (4)	−0.0419 (4)	0.14958 (18)	0.0349 (10)	
H9A	0.2570	−0.0290	0.1905	0.042*	
H9B	0.2618	−0.1197	0.1360	0.042*	
C10	0.0478 (5)	−0.0518 (4)	0.15017 (18)	0.0342 (10)	
C11	0.3746 (4)	0.0071 (4)	0.06312 (18)	0.0325 (9)	
H11A	0.4592	−0.0398	0.0801	0.039*	
H11B	0.4168	0.0745	0.0402	0.039*	
C12	0.2791 (5)	−0.0758 (4)	0.02112 (18)	0.0327 (9)	
C13	0.2498 (4)	0.1765 (4)	−0.06094 (19)	0.0378 (10)	
H13	0.2383	0.0963	−0.0746	0.045*	
C14	0.3463 (5)	0.2557 (4)	−0.0924 (2)	0.0452 (11)	
H14	0.3974	0.2285	−0.1262	0.054*	
C15	0.3644 (5)	0.3735 (4)	−0.0725 (2)	0.0466 (12)	
H15	0.4320	0.4259	−0.0918	0.056*	
C16	0.2812 (5)	0.4161 (4)	−0.02308 (19)	0.0381 (10)	
C17	0.1868 (4)	0.3301 (4)	0.00554 (18)	0.0317 (9)	
C18	0.0961 (4)	0.3654 (4)	0.05541 (18)	0.0336 (10)	
C19	0.0966 (5)	0.4885 (4)	0.0753 (2)	0.0378 (10)	
C20	0.1934 (5)	0.5738 (4)	0.0444 (2)	0.0487 (12)	
H20	0.1951	0.6554	0.0568	0.058*	
C21	0.2807 (5)	0.5397 (4)	−0.0012 (2)	0.0465 (12)	
H21	0.3430	0.5978	−0.0193	0.056*	
C22	0.0007 (5)	0.5157 (4)	0.1236 (2)	0.0452 (11)	
H22	−0.0044	0.5955	0.1383	0.054*	
C23	−0.0846 (5)	0.4261 (4)	0.1489 (2)	0.0475 (12)	
H23	−0.1471	0.4445	0.1812	0.057*	
C24	−0.0788 (5)	0.3064 (4)	0.12670 (19)	0.0407 (10)	
H24	−0.1386	0.2462	0.1445	0.049*	

### Atomic displacement parameters (Å^2^)

**Table:**

	*U*^11^	*U*^22^	*U*^33^	*U*^12^	*U*^13^	*U*^23^
Cu1	0.0304 (3)	0.0260 (3)	0.0346 (3)	−0.0016 (2)	0.0050 (2)	0.0013 (2)
O5	0.0316 (15)	0.0351 (16)	0.0403 (17)	0.0001 (13)	0.0064 (13)	0.0070 (13)
O7	0.0301 (16)	0.0294 (15)	0.0383 (16)	−0.0020 (12)	0.0024 (12)	−0.0052 (12)
N1	0.0304 (18)	0.0289 (18)	0.0320 (18)	−0.0030 (15)	0.0074 (14)	0.0003 (15)
N2	0.0305 (18)	0.0294 (18)	0.0358 (19)	0.0004 (15)	−0.0009 (15)	0.0044 (15)
N3	0.0286 (18)	0.0318 (19)	0.039 (2)	−0.0017 (15)	0.0023 (15)	0.0029 (15)
O6	0.0423 (18)	0.0338 (17)	0.062 (2)	−0.0010 (14)	0.0179 (15)	−0.0130 (15)
O2	0.064 (2)	0.053 (2)	0.0415 (19)	0.0204 (17)	0.0086 (16)	0.0072 (16)
O3	0.048 (2)	0.0419 (18)	0.075 (2)	0.0052 (16)	−0.0136 (17)	−0.0256 (17)
O1	0.072 (2)	0.060 (2)	0.050 (2)	0.0073 (19)	0.0139 (18)	0.0041 (18)
O4	0.0411 (18)	0.0484 (19)	0.061 (2)	−0.0050 (15)	0.0101 (15)	0.0244 (16)
C1	0.053 (3)	0.033 (2)	0.044 (3)	−0.003 (2)	−0.002 (2)	−0.007 (2)
C2	0.064 (3)	0.044 (3)	0.056 (3)	0.013 (2)	−0.016 (3)	−0.018 (2)
C3	0.038 (2)	0.034 (2)	0.048 (3)	0.002 (2)	−0.011 (2)	−0.008 (2)
C4	0.037 (2)	0.059 (3)	0.049 (3)	−0.001 (2)	0.004 (2)	−0.022 (2)
C5	0.034 (2)	0.053 (3)	0.044 (3)	−0.013 (2)	0.0040 (19)	−0.011 (2)
C6	0.024 (2)	0.031 (2)	0.032 (2)	−0.0003 (17)	−0.0007 (16)	0.0014 (18)
C7	0.032 (2)	0.032 (2)	0.037 (2)	0.0009 (18)	0.0025 (18)	0.0010 (18)
C8	0.039 (2)	0.029 (2)	0.045 (3)	−0.0052 (19)	−0.003 (2)	0.004 (2)
C9	0.033 (2)	0.037 (2)	0.035 (2)	−0.0031 (18)	0.0012 (18)	0.0068 (19)
C10	0.035 (2)	0.035 (2)	0.033 (2)	−0.005 (2)	0.0054 (18)	0.0002 (19)
C11	0.025 (2)	0.031 (2)	0.042 (2)	0.0012 (17)	0.0034 (17)	0.0002 (18)
C12	0.033 (2)	0.025 (2)	0.040 (2)	−0.0044 (18)	0.0132 (18)	0.0025 (18)
C13	0.033 (2)	0.039 (2)	0.042 (2)	−0.0031 (19)	0.0045 (19)	0.004 (2)
C14	0.041 (3)	0.050 (3)	0.045 (3)	0.003 (2)	0.008 (2)	0.011 (2)
C15	0.033 (2)	0.050 (3)	0.056 (3)	−0.006 (2)	0.005 (2)	0.016 (2)
C16	0.035 (2)	0.032 (2)	0.047 (3)	−0.0062 (19)	−0.009 (2)	0.014 (2)
C17	0.027 (2)	0.030 (2)	0.038 (2)	−0.0023 (17)	−0.0051 (17)	0.0051 (19)
C18	0.031 (2)	0.032 (2)	0.037 (2)	−0.0028 (18)	−0.0089 (18)	0.0046 (18)
C19	0.036 (2)	0.031 (2)	0.046 (3)	−0.0014 (19)	−0.0115 (19)	0.001 (2)
C20	0.057 (3)	0.030 (2)	0.059 (3)	−0.010 (2)	−0.017 (3)	0.002 (2)
C21	0.044 (3)	0.038 (3)	0.058 (3)	−0.013 (2)	−0.006 (2)	0.009 (2)
C22	0.045 (3)	0.035 (3)	0.056 (3)	0.002 (2)	−0.011 (2)	−0.009 (2)
C23	0.050 (3)	0.049 (3)	0.044 (3)	0.010 (2)	−0.002 (2)	−0.009 (2)
C24	0.038 (2)	0.042 (3)	0.042 (3)	0.002 (2)	0.003 (2)	0.002 (2)

### Geometric parameters (Å, °)

**Table:**

Cu1—O7	1.987 (3)	C5—H5	0.9300
Cu1—O5	1.997 (3)	C6—C7	1.387 (5)
Cu1—N2	2.003 (3)	C7—C8	1.397 (6)
Cu1—N3	2.049 (3)	C7—H7	0.9300
Cu1—O7^i^	2.293 (3)	C8—H8	0.9300
Cu1—N1	2.460 (3)	C9—C10	1.514 (5)
O5—C10	1.272 (5)	C9—H9A	0.9700
O7—C12	1.290 (5)	C9—H9B	0.9700
O7—Cu1^i^	2.293 (3)	C11—C12	1.536 (5)
N1—C6	1.420 (5)	C11—H11A	0.9700
N1—C11	1.462 (5)	C11—H11B	0.9700
N1—C9	1.465 (5)	C13—C14	1.401 (6)
N2—C24	1.325 (5)	C13—H13	0.9300
N2—C18	1.366 (5)	C14—C15	1.364 (6)
N3—C13	1.327 (5)	C14—H14	0.9300
N3—C17	1.363 (5)	C15—C16	1.406 (6)
O6—C12	1.219 (4)	C15—H15	0.9300
O2—C1	1.333 (5)	C16—C17	1.406 (5)
O2—H2	0.8200	C16—C21	1.431 (6)
O3—C3	1.392 (5)	C17—C18	1.426 (5)
O3—C2	1.422 (5)	C18—C19	1.411 (6)
O1—C1	1.201 (5)	C19—C22	1.405 (6)
O4—C10	1.252 (5)	C19—C20	1.437 (6)
C1—C2	1.510 (6)	C20—C21	1.331 (6)
C2—H2A	0.9700	C20—H20	0.9300
C2—H2B	0.9700	C21—H21	0.9300
C3—C4	1.376 (6)	C22—C23	1.354 (6)
C3—C8	1.382 (6)	C22—H22	0.9300
C4—C5	1.381 (6)	C23—C24	1.395 (6)
C4—H4	0.9300	C23—H23	0.9300
C5—C6	1.393 (5)	C24—H24	0.9300
			
O7—Cu1—O5	92.08 (11)	C3—C8—C7	120.5 (4)
O7—Cu1—N2	171.29 (12)	C3—C8—H8	119.8
O5—Cu1—N2	93.61 (12)	C7—C8—H8	119.8
O7—Cu1—N3	91.96 (12)	N1—C9—C10	115.5 (3)
O5—Cu1—N3	170.40 (12)	N1—C9—H9A	108.4
N2—Cu1—N3	81.44 (13)	C10—C9—H9A	108.4
O7—Cu1—O7^i^	77.11 (11)	N1—C9—H9B	108.4
O5—Cu1—O7^i^	93.01 (10)	C10—C9—H9B	108.4
N2—Cu1—O7^i^	109.13 (11)	H9A—C9—H9B	107.5
N3—Cu1—O7^i^	96.38 (11)	O4—C10—O5	123.8 (4)
O7—Cu1—N1	74.69 (11)	O4—C10—C9	116.0 (4)
O5—Cu1—N1	76.84 (11)	O5—C10—C9	120.2 (3)
N2—Cu1—N1	100.21 (12)	N1—C11—C12	111.2 (3)
N3—Cu1—N1	95.87 (12)	N1—C11—H11A	109.4
O7^i^—Cu1—N1	149.54 (10)	C12—C11—H11A	109.4
C10—O5—Cu1	119.6 (2)	N1—C11—H11B	109.4
C12—O7—Cu1	120.4 (2)	C12—C11—H11B	109.4
C12—O7—Cu1^i^	134.3 (2)	H11A—C11—H11B	108.0
Cu1—O7—Cu1^i^	102.89 (11)	O6—C12—O7	124.5 (4)
C6—N1—C11	119.6 (3)	O6—C12—C11	119.4 (4)
C6—N1—C9	115.8 (3)	O7—C12—C11	116.1 (3)
C11—N1—C9	111.7 (3)	N3—C13—C14	122.9 (4)
C6—N1—Cu1	108.0 (2)	N3—C13—H13	118.5
C11—N1—Cu1	96.4 (2)	C14—C13—H13	118.5
C9—N1—Cu1	101.4 (2)	C15—C14—C13	119.0 (4)
C24—N2—C18	118.2 (4)	C15—C14—H14	120.5
C24—N2—Cu1	128.9 (3)	C13—C14—H14	120.5
C18—N2—Cu1	112.4 (3)	C14—C15—C16	120.3 (4)
C13—N3—C17	117.8 (3)	C14—C15—H15	119.9
C13—N3—Cu1	130.4 (3)	C16—C15—H15	119.9
C17—N3—Cu1	111.3 (3)	C17—C16—C15	116.6 (4)
C1—O2—H2	109.5	C17—C16—C21	117.8 (4)
C3—O3—C2	117.1 (4)	C15—C16—C21	125.5 (4)
O1—C1—O2	124.9 (4)	N3—C17—C16	123.2 (4)
O1—C1—C2	125.0 (4)	N3—C17—C18	116.1 (3)
O2—C1—C2	110.1 (4)	C16—C17—C18	120.7 (4)
O3—C2—C1	112.3 (4)	N2—C18—C19	123.1 (4)
O3—C2—H2A	109.1	N2—C18—C17	116.9 (4)
C1—C2—H2A	109.1	C19—C18—C17	120.0 (4)
O3—C2—H2B	109.1	C22—C19—C18	116.2 (4)
C1—C2—H2B	109.1	C22—C19—C20	126.3 (4)
H2A—C2—H2B	107.9	C18—C19—C20	117.6 (4)
C4—C3—C8	119.2 (4)	C21—C20—C19	122.1 (4)
C4—C3—O3	124.2 (4)	C21—C20—H20	118.9
C8—C3—O3	116.5 (4)	C19—C20—H20	118.9
C3—C4—C5	120.2 (4)	C20—C21—C16	121.7 (4)
C3—C4—H4	119.9	C20—C21—H21	119.1
C5—C4—H4	119.9	C16—C21—H21	119.1
C4—C5—C6	121.9 (4)	C23—C22—C19	120.3 (4)
C4—C5—H5	119.1	C23—C22—H22	119.8
C6—C5—H5	119.1	C19—C22—H22	119.8
C7—C6—C5	117.4 (4)	C22—C23—C24	120.1 (4)
C7—C6—N1	123.3 (3)	C22—C23—H23	119.9
C5—C6—N1	119.2 (3)	C24—C23—H23	119.9
C6—C7—C8	120.8 (4)	N2—C24—C23	122.1 (4)
C6—C7—H7	119.6	N2—C24—H24	119.0
C8—C7—H7	119.6	C23—C24—H24	119.0

Symmetry codes: (i) −*x*, −*y*, −*z*.

### Hydrogen-bond geometry (Å, °)

**Table:**

*D*—H···*A*	*D*—H	H···*A*	*D*···*A*	*D*—H···*A*
O2—H2···O4^ii^	0.82	1.82	2.622 (4)	164

Symmetry codes: (ii) *x*, *y*+1, *z*.
